# Draft Genome Sequence of *Desulfitobacterium hafniense* Strain DH, a Sulfate-Reducing Bacterium Isolated from Paddy Soils

**DOI:** 10.1128/genomeA.01693-15

**Published:** 2016-02-11

**Authors:** Xi Zhang, Guo-Xiang Li, Song-Can Chen, Xiao-Yu Jia, Kun Wu, Chang-Li Cao, Peng Bao

**Affiliations:** a Key Laboratory of Urban Environment and Health, Institute of Urban Environment, Chinese Academy of Sciences, Xiamen, People’s Republic of China; b Ningbo Urban Environment Observation and Station, Chinese Academy of Sciences, Ningbo, People’s Republic of China; c State Key Lab of Urban and Regional Ecology, Research Center for Eco-Environmental Sciences, Chinese Academy of Sciences, Beijing, People’s Republic of China

## Abstract

*Desulfitobacterium hafniense* strain DH is a sulfate-reducing species. Here, we report the draft genome sequence of strain DH, with a size of 5,368,588 bp, average G+C content of 47.48%, and 5,296 predicted protein-coding sequences.

## GENOME ANNOUNCEMENT

*Desulfitobacterium hafniense* is an anaerobic spore-forming microorganism. Its type strain is Co23 (=ATCC 700175 = DSM 11544). It is notable for being capable of reductive dechlorination of organochloride (1). When grown on pyruvate, *Desulfitobacterium hafniense* produces sulfide if thiosulfate or sulfite is added as an electron acceptor. Fe(III) is reduced to Fe(II), but Mn(IV) is not reduced. Sulfate is not reduced to sulfide in the presence of pyruvate or other carbon sources typically used by sulfate-reducing bacteria (SRB). The whole genome of *Desulfitobacterium hafniense* strain Y51 was recently completed (2). The partial genome sequence of strain DCB-2 has been available since 2001 (1) at the Joint Genome Institute (http://www.jgi.doe.gov). We performed draft-genome sequencing of *Desulfitobacterium hafniense* strain DH, which was isolated from paddy soil in Yunnan province, China. Not like traditional *Desulfitobacterium hafniense*, the strain DH can reduce sulfate in the presence of acetate and yeast extract, which confirms that it is a sulfate-reducing bacterium.

Isolation of genomic DNA was carried out using the SDS method. Total DNA obtained was subjected to quality control by agarose gel electrophoresis and quantified by Qubit. The genome of *Desulfitobacterium hafniense* strain DH was sequenced with MPS (massively parallel sequencing) Illumina technology. Two DNA libraries were constructed: a paired-end library with an insert size of 500 bp and a mate-pair library with an insert size of 5 kb. The 500-bp library and the 5-kb library were sequenced using an Illumina HiSeq2500 by PE125 strategy. Quality control of both paired-end and mate-pair reads were performed using an in-house program. After this step, Illumina PCR adapter reads and low-quality reads were filtered. The filtered reads were assembled by SOAPdenovo (3, 4) (http://soap.genomics.org.cn/soapdenovo.html) to generate scaffolds. tRNA genes were predicted with tRNAscan-SE (5), rRNA genes were predicted with rRNAmmer (6), and sRNAs were predicted by BLAST against the Rfam (7) database. Repetitive sequences were predicted using RepeatMasker (8). Tandem repeats were analyzed using Tandem Repeats Finder (9). PHAST (10) was used for prophage prediction, and CRISPRFinder (11) was used for CRISPR identification. Gene prediction was performed on the *Desulfitobacterium hafniense* strain DH genome assembly by GeneMarkS (12) (http://topaz.gatech.edu) with an integrated model that combined the GeneMarkS generated (native) and Heuristic model parameters.

This resulted in a draft genome consisting of 104 scaffolds and 114 contigs for a total of 5,368,588 bp and a G+C content of 47.48%. The genome was shown to encode at least 61 predicted RNAs, including 2 rRNAs, 54 tRNAs, and 5 sRNAs. A total of 5,296 identified genes yielded a coding capacity of 4,677,153 bp (coding percentage, 87.12%). A genome-wide comparison showed 86.98% coverage and 94.5% similarity with the complete genome of *Desulfitobacterium hafniense* Y51 (accession no. GCF_000010055.1) (2). These data confirm *Desulfitobacterium hafniense* strain DH as a unique species within the *D. hafniense* complex.

### Nucleotide sequence accession numbers.

The *Desulfitobacterium hafniense* strain DH genome sequence has been deposited at DDBJ/EMBL/GenBank under the accession number LOCK00000000. The version described in this paper is the first version, LOCK01000000.
